# Supplementary material for: Matrix Metalloproteinases on Severe COVID-19 Lung Disease Pathogenesis: Cooperative Actions of MMP-8/MMP-2 Axis on Immune Response through HLA-G Shedding and Oxidative Stress
Source: Biomolecules. 2022 Apr 19;12(5):604. doi: 10.3390/biom12050604 (PMC9138255; doi:10.3390/biom12050604)
Supplement: Supplementary file 1 [file biomolecules-12-00604-s001.zip › biomolecules-1649666-supplementary.pdf]

Supplementary Materials

Original article: "Matrix Metalloproteinases on Severe COVID-19 Lung Disease Pathogenesis: Cooperative Actions of MMP-8/MMP-2 Axis on Immune Response Through HLA-G Shedding and Oxidative Stress"

Supplementary Figure S1 - Initial Screening of TAF (tracheal aspirate fluid) in zymogram. Three different protein concentrations of three patients, chosen at random, were applied per lane in **(A)** gelatin and **(B)** casein zymograms.

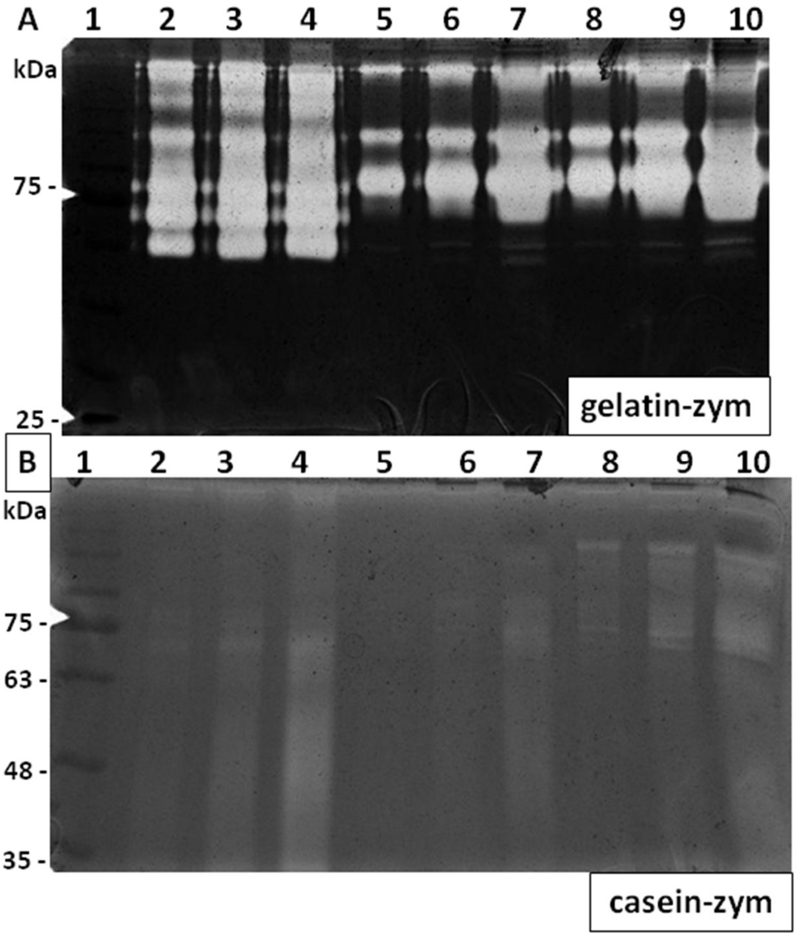

A: Twelve percent gelatin zymogram stained with Coomassie Brilliant Blue. Lane 1: Prestained BluEye Protein Ladder (Sigma), with 75- and 25-kDa markers indicated based on cuts made on the gel prior to staining with Coomassie Brilliant Blue. Lanes 2-4: (non-COVID-19), Lanes 5-7: patient Survival COVID-19, Lanes 8-10: patient Non-survival COVID-19 (0.1; 0.3; 0.5 ug of protein), respectively. **B:** Twelve percent casein zymogram stained with Coomassie Brilliant Blue. The staining is fainter than the zymogram in A, as casein usually stains less. Exactly the same amounts of total protein from TAF from the same patients loaded in the same gel A lanes were applied.

Supplementary Figure S2 - Initial Screening of TAF in zymogram regarding the main class of protease present in patient samples. Two different protein concentrations of three patients were applied per lane in gelatin zymogram. **(A)** Zymogram was incubated with the regular Tris-HCl/$CaCl_2$ buffer (the zymogram buffer), while gels **(B)** were incubated overnight with 1 mM Phenanthroline and **(C)** were incubated with 1mM PMSF, respectively.

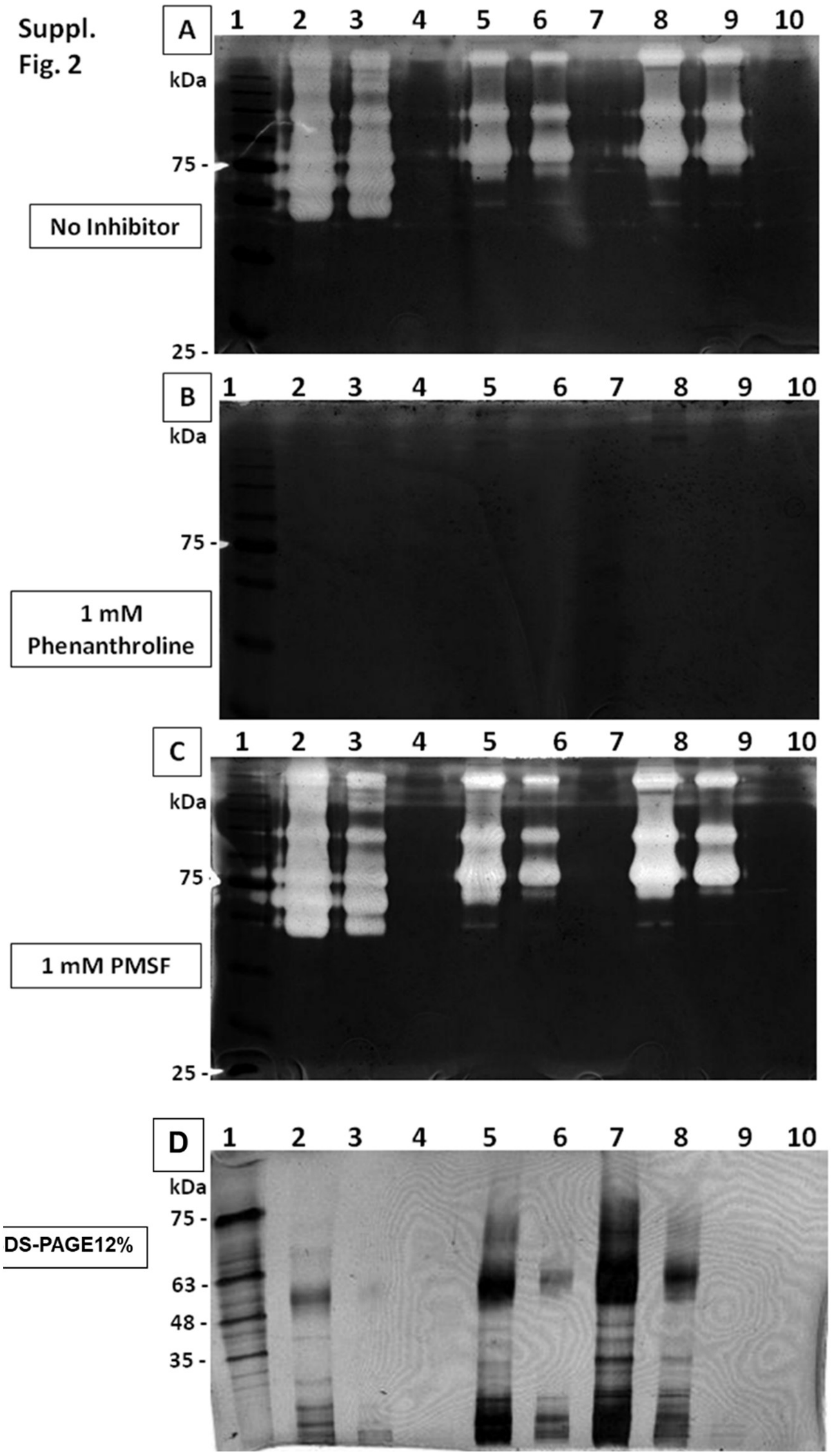

A: Twelve percent gelatin zymogram stained with Coomassie Brilliant Blue after overnight incubation in Tris-HCl/$CaCl_2$ buffer (the zymogram buffer). Lane 1: Prestained BluEye Protein Ladder (Sigma), with 75- and 25-kDa markers indicated based on cuts made on the gel prior to staining with Coomassie Brilliant Blue. Lanes 2-3: (0.05 ug; 0.15 ug of protein) Non-COVID-19, Lanes 5-6: (0.05 ug; 0.15 ug of protein) patient Survival COVID-19, Lanes 8-9: patient Non-survival COVID-19 (0.05; 0.15; ug of protein), Lanes 4, 7,10 empty respectively. **B:** Twelve percent gelatin zymogram stained with Coomassie Brilliant Blue. The whole gel was incubated overnight in the zymogram buffer containing 1 mM Phenanthroline. The samples and protein masses loaded are equal to gel A. **C:** Twelve percent gelatin zymogram stained with Coomassie Brilliant Blue. The whole gel was incubated overnight in the zymogram buffer containing 1 mM Phenyl-methyl-sulphonylfluoride (PMSF) at 1 mM. The samples and protein masses loaded are equal to gel A. **D**: Twelve percent SDS-PAGE gel stained with silver nitrate. Lanes 2-3: Non-COVID-19, Lanes 5-6: patient Survival COVID-19, Lanes 7-8: patient Non-survival COVID-19 (10 ug of protein), Lanes 4, 9,10 empty respectively.

Supplementary Figure S3. Representative gelatin zymogram of TAF samples from COVID-19 patients and non-COVID-19 controls.

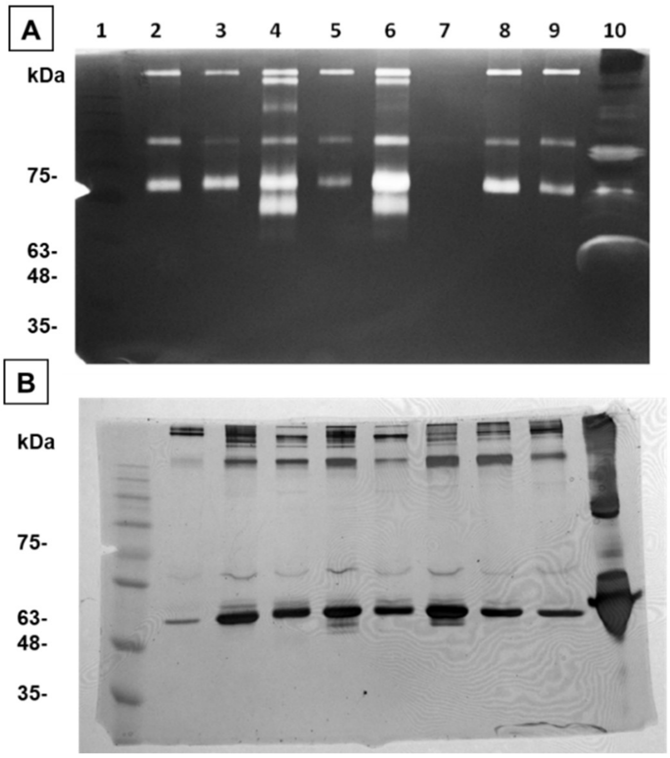

A: Twelve percent SDS-PAGE gel stained with Coomassie Brilliant Blue. Lane 1: Prestained BluEye Protein Ladder (Sigma). Lanes 2 and 3: two control patients (non-COVID-19) who were in the same hospital but were negative for COVID19. Lanes 4-7: samples from 4 critical SARS-COV-2-infected patients that died (Non-survival). Lanes 8-9: 2 samples from 2 critical SARS-COV-2-infected patients that survived (survival) (1 ug of protein). Lane 10: Fetal Bovine Serum. **B:** Twelve percent SDS-PAGE gel stained with silver nitrate. Lanes 2 and 3: two control patients (non-COVID-19) who were in the same hospital but were negative for COVID19. Lanes 4-7: samples of 4 critical patients infected with SARS-COV2 who died (Non-survival). Lanes 8-9: 2 samples from 2 critical SARS-COV2-infected patients that survived (Survival) (10 ug of protein). Lane 10: Fetal Bovine Serum.

Supplementary Figure S4. Cell differentiation in TAF from non-COVID-19 and COVID-19 patients.

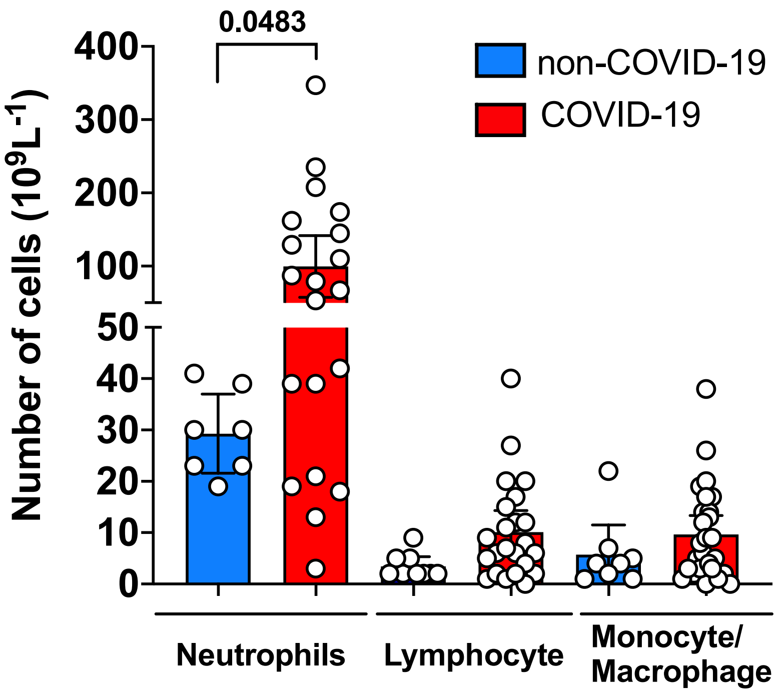

Absolute neutrophil, Lymphocyte and monocyte/ macrophage counts in TAF samples from non-COVID-19 and COVID-19 patients. Statistical analyzes were performed using the Kruskal-Wallis multiple comparison test, followed by the Dunns post-test to compare pairs. Data are expressed as median with 95% confidence intervals. Statistical differences between groups are considered by $p < 0.05$ and represented direct in the graphic figure.

Supplementary Table S1. Description of pre-existing diseases in non-COVID-19 patients

Baseline Variable	non-COVID-19 N= 13
**Base disease No. (%)**	
Brain aneurysm	2 (15.3)
CA Brain	1 (7.7)
Pancreatitis	1(7.7)
Nephropathy	1(7.7)
DPOC	1(7.7)
Others	7 (53.8)

CA- Cancer, DPOC - Chronic obstructive pulmonary disease

Supplementary Table S2. Statistical information about *p*-value of proteomics reanalyzes

Uniprot ID	Gene name	t	df	*p* value	*p*.adj
A0A0U1RRL7	MMP24OS	-0.346208644	7.628149286	0.738540407	0.846135094
O75900	MMP23B	1.451850712	6.237048395	0.194919826	0.373242517
P03956	MMP1	-2.049951361	6.608866347	0.081918071	0.215555977
P08253	MMP2	-2.124596412	15.76382051	0.049799151	0.158060835
P09237	MMP7	-2.229832893	13.83080463	0.042860756	0.142757009
P09238	MMP10	-1.75417077	9.010757184	0.113257492	0.26319364
P14780	MMP9	-0.978116457	30.19615568	0.335788517	0.524411715
P22894	MMP8	-2.077762291	26.05263093	0.047724825	0.153479323
P39900	MMP12	1.817928401	7.998976205	0.106594355	0.253771147
P50281	MMP14	-5.382421924	26.68595537	1.13231E-05	0.00050751
P51511	MMP15	3.089793801	6.098825536	0.020946559	0.090643843
Q99542	MMP19	-0.959149878	7.441432919	0.367604031	0.554444983
Q9H239	MMP28	0.519408714	5.91404534	0.622333043	0.767059358
